# Intrathoracic Vertebral Allograft Migration After Surgery for Pediatric Pott Disease: A Case Report With Radiologic-Pathologic Correlation and a Critical Diagnostic Review

**DOI:** 10.7759/cureus.110996

**Published:** 2026-06-16

**Authors:** José A García, Luis F Manzano Romero, Juan Eduardo Sebastián Aguirre Garza, Gerardo E Muñoz-Maldonado, Marco A Ponce Camacho, Andres Almaguer Acevedo

**Affiliations:** 1 General Surgery, Hospital Universitario “Dr. José Eleuterio González”, Monterrey, MEX; 2 Pathological Anatomy and Cytopathology, Hospital Universitario “Dr. José Eleuterio González”, Monterrey, MEX

**Keywords:** diagnostic thoracotomy, intrathoracic migration, pediatric spinal tuberculosis, pleural fibrosis, pott disease, tuberculous pleurisy, vertebral allograft, video-assisted thoracoscopic surgery

## Abstract

Pediatric Pott disease can require debridement, corpectomy, and anterior column reconstruction when vertebral destruction, instability, deformity, or neurologic risk is present. Years later, postoperative structural sequelae may overlap clinically and radiologically with suspected recurrent thoracic tuberculosis. We report the case of a six-year-old girl with thoracic vertebral tuberculosis diagnosed in 2021, supported by immunologic, molecular, and histopathologic evidence, including vertebral granulomatous inflammation with acid-fast bacilli. She underwent right posterolateral thoracotomy, thoracic corpectomy centered at T4, and reconstruction with an approximately 2-cm fibular structural allograft, followed by completion of antituberculous therapy.

In 2025, she presented with fever, persistent cough, recurrent respiratory episodes, weight loss, severe malnutrition, calcified mediastinal findings, extrinsic airway compression, and intrathoracic bone-density material adjacent to the prior reconstruction, raising concern for pulmonary reinfection or pleural tuberculosis. GeneXpert, acid-fast bacilli smears, cultures, and mycobacterial polymerase chain reaction testing were negative or non-confirmatory, and no safe percutaneous biopsy window was documented.

Diagnostic right posterolateral thoracotomy demonstrated pleuropulmonary adhesions and material compatible with a migrated fibular allograft, without abnormal thoracic or hilar lymph nodes. Right pleural histopathology showed fibrosing pleuritis without granulomas, caseous necrosis, active inflammation, acid-fast bacilli, or malignancy. Empirical antituberculous retreatment was not restarted. This case, accompanied by a focused critical diagnostic review, emphasizes multimodal correlation to differentiate active tuberculous relapse from postoperative structural mimics.

## Introduction

Treatment and retreatment decisions in pediatric tuberculosis (TB) require integration of disease severity, likelihood of microbiologic confirmation, drug-susceptibility concerns, and the clinical risk of delayed therapy [1-3]. Spinal TB, or Pott disease, is a destructive form of musculoskeletal TB that may produce vertebral collapse, paraspinal extension, deformity, instability, and neurologic compromise [4,5]. In children, residual growth and spinal flexibility increase the long-term relevance of postinfectious kyphosis and mechanical sequelae [6,7].

Most children with spinal TB are managed medically, but surgery may be required in the presence of severe vertebral destruction, progressive deformity, instability, neurologic compromise, extensive abscesses, or failure of medical treatment [1,4-8]. Reconstructive options include structural autograft or allograft, fibular strut graft, cage-based reconstruction, and instrumented stabilization [8-11]. Although these techniques restore anterior column support, graft-related migration is a recognized mechanical complication in spine surgery [12,13].

In suspected thoracic tuberculous relapse after prior spinal reconstruction, less-invasive diagnostic strategies should be considered before open thoracotomy whenever they are safe and technically feasible. These may include image-guided biopsy or aspiration, pleural biopsy, medical thoracoscopy, or video-assisted thoracoscopic surgery (VATS), depending on target accessibility, anatomy, adhesions, airway risk, and local expertise [14-19]. Functional imaging such as positron emission tomography-computed tomography may be useful as an adjunct in selected settings, but it does not replace tissue correlation when active infection, malignancy, and postoperative inflammatory change remain competing explanations [20].

This report describes a child with tissue-supported thoracic Pott disease treated with corpectomy and fibular allograft reconstruction who later developed respiratory symptoms and thoracic imaging findings concerning for pulmonary reinfection or pleural TB. The central diagnostic lesson is that prior TB, constitutional symptoms, calcified thoracic findings, and distorted postoperative anatomy can create anchoring bias. The case is therefore presented as a case report with radiologic-pathologic correlation and a critical diagnostic review focused on differentiating active tuberculous relapse from structural postoperative mimics.

## Case presentation

A six-year-old girl had a history of thoracic vertebral TB diagnosed in 2021. The original diagnosis was not treated on a simple clinical suspicion: the available documentation reported a positive QuantiFERON test, molecular detection reported as MTB/NTM (*Mycobacterium tuberculosis*/nontuberculous mycobacteria) in a vertebral lesion, and vertebral histopathology showing chronic granulomatous inflammation associated with acid-fast bacilli on Ziehl-Neelsen staining. A prior computed tomography (CT)-guided Tru-Cut biopsy from the T3-T4 region showed fibrocartilaginous tissue negative for neoplasia, while the later vertebral specimen provided etiologic support for Pott disease.

In 2021, she underwent right posterolateral thoracotomy, debridement, thoracic corpectomy centered at T4, and reconstruction with an approximately 2-cm structural fibular allograft. Source documents contain level discrepancies, including T2-T4/T3-T4; therefore, the level is described conservatively as centered at T4. Estimated intraoperative blood loss was approximately 200 cc; transfusion was required, and the postoperative course included pediatric intensive care unit admission and transient mechanical ventilation. She subsequently completed a documented 12-month antituberculous regimen described in the source material as DOTBAL/RIPE-type therapy and was discharged from TB treatment in 2022.

A condensed chronology is clinically relevant: the 2021 episode established vertebral TB and required reconstruction; antituberculous therapy was completed in 2022; respiratory episodes with elevated inflammatory markers were documented in 2024 and early 2025; the October-November 2025 presentation raised concern for pulmonary reinfection or pleural TB; and diagnostic thoracotomy on November 14, 2025, clarified the operative and pathologic diagnosis.

In 2025, she was readmitted with fever, persistent cough, recurrent respiratory episodes or pneumonias, hyporexia, weight loss, and severe malnutrition. Physical examination documented thoracic kyphotic deformity/gibbus and low weight. No peripheral pathologic lymphadenopathy was documented in the reviewed material. The Infectious Disease team considered the working diagnosis to be probable pulmonary TB reinfection versus pleural TB without microbiologic confirmation. The available documentation did not establish a single cause for fever, weight loss, or severe malnutrition; these findings were therefore interpreted as clinically important but non-specific constitutional features rather than as proof of active TB, isolated airway compression, or a single nutritional diagnosis.

Inflammatory markers varied across documented respiratory episodes and supported systemic inflammation without establishing a specific etiology (Table 1).

**Table 1:** Inflammatory markers across documented respiratory episodes CRP: C-reactive protein; ESR: Erythrocyte sedimentation rate; WBC: White blood cell count; uL: microliter. Not documented indicates that the value was not available in the reviewed source documentation for that episode. Reference intervals may vary by laboratory.

Parameters	February 2024 respiratory episode	December 2024 respiratory episode	January 2025 respiratory/infectious follow-up	October 2025 respiratory episode
CRP (mg/L)	60	176	47	132
CRP reference range (mg/L)	0-10	0-10	0-10	0-10
ESR (mm/h)	Not documented	Not documented	42	53
ESR reference range (mm/h)	0-20	0-20	0-20	0-20
WBC (x10^3^/uL)	7.94	12	6.66	16.9
WBC reference range (x10^3^/uL)	4.00-11.00	4.00-11.00	4.00-11.00	4.00-11.00
Interpretation/clinical context	Elevated CRP with WBC within the local reference interval; inflammatory but non-specific.	Marked CRP elevation with mild leukocytosis; inflammatory but non-specific.	CRP and ESR elevated with WBC within the local reference interval.	CRP and ESR elevated with leukocytosis, supporting systemic inflammation without specifying etiology.

**Figure 1:**
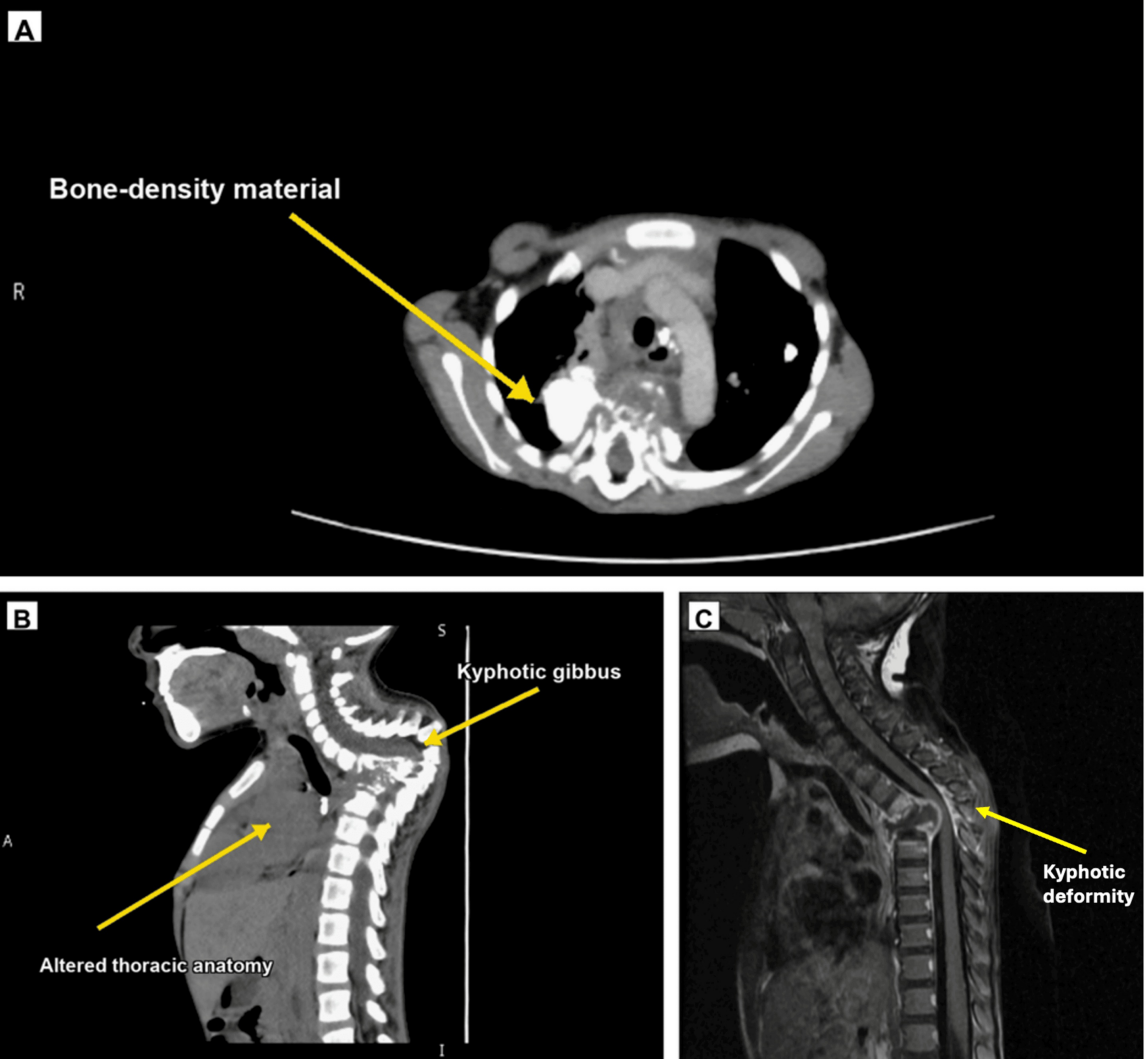
Contrast-enhanced computed tomography and magnetic resonance imaging correlation of intrathoracic bone-density material and chronic postoperative thoracic deformity (A) Axial contrast-enhanced chest computed tomography demonstrates right intrathoracic/paravertebral bone-density material adjacent to the prior thoracic corpectomy/reconstruction region (arrow). (B) Sagittal contrast-enhanced computed tomography reconstruction demonstrates dorsal kyphotic deformity and altered thoracic anatomy (arrows). (C) Sagittal T2-weighted magnetic resonance imaging demonstrates chronic cervicothoracic/upper thoracic kyphotic deformity and postoperative/postinfectious structural alteration (arrow). The computed tomography panels are contrast-enhanced; the magnetic resonance image is not interpreted as a contrast-enhanced sequence. These images document an anatomic substrate for a structural mimic and should not be interpreted alone as proof of active tuberculous recurrence.

Thoracic imaging documented an approximately 46-degree dorsal kyphotic gibbus, calcified mediastinal lymph nodes and/or a calcified granuloma, and an approximately 24 x 22 mm intrathoracic bone-density structure adjacent to the previous thoracic reconstruction region, interpreted as compatible with migrated allograft material in the appropriate clinical context. Specific signs supporting allograft migration included the bone-density character of the lesion, its relationship to the prior corpectomy/reconstruction region, lack of an abnormal nodal target at surgery, and operative visualization of extrinsic material compatible with prior bone graft. The same postoperative anatomy also complicated safe sampling access and contributed to the differential diagnosis of calcified postinfectious sequelae, active granulomatous disease, malignancy, or migrated allograft material (Figure 1).

The bronchoscopy report documented extrinsic tracheobronchial compression and distorted airway anatomy, without an ulcerated, necrotic, or granulomatous endoluminal lesion suggestive of active endobronchial TB. The bronchoscopy was complicated by severe bronchospasm. Microbiologic evaluation in 2025 did not confirm active TB: available GeneXpert/Xpert MTB/RIF (*Mycobacterium tuberculosis*/rifampicin resistance) ultra testing, AFB (acid-fast bacilli) smears, cultures, and mycobacterial PCR were negative or non-confirmatory in the reviewed documentation. Bronchial lavage documentation also reported no bacterial morphotypes on Gram stain, negative aerobic bacterial culture, negative KOH (potassium hydroxide) preparation, negative fungal culture, and no detection of *Coccidioides immitis*. The final mycobacterial culture incubation duration was not documented in the reviewed record. No safe percutaneous biopsy window was documented.

No PET-CT study or alternative safely accessible image-guided biopsy target was documented in the reviewed record. Because the diagnostic uncertainty had major therapeutic consequences and there was no documented safe percutaneous biopsy route, diagnostic right posterolateral thoracotomy was performed on November 14, 2025. The structured operative record included a respiratory tuberculosis-related administrative diagnostic entry without documented microbiologic confirmation; this was interpreted as contextual documentation of clinical suspicion rather than evidence of active tuberculosis. The operative narrative described right pleural entry, pleuropulmonary adhesions, an upper lobe adherent to the pleura, and extrinsic material compatible with prior bone graft. No abnormal thoracic or hilar lymph nodes were identified, and the specimen submitted for pathology was right pleura rather than lymph node tissue.

Histopathology of the right pleura showed fibrosing pleuritis/pleural fibrosis. No granulomas, caseous necrosis, active inflammation, AFB, or malignant cells were identified. These findings did not support active pleural TB in 2025. The final integrated diagnosis was late intrathoracic migration of vertebral allograft material after pediatric Pott disease surgery, with fibrosing pleuritis and clinicoradiologic simulation of pulmonary reinfection or pleural TB. Empirical antituberculous retreatment was not restarted after pathology.

The 2025 suspicion of pulmonary reinfection or pleural TB was clinically reasonable: the patient had prior confirmed TB, fever, persistent cough, weight loss, recurrent respiratory episodes, elevated inflammatory markers, calcified thoracic findings, and altered mediastinal/thoracic anatomy. However, suspicion alone was not sufficient to justify automatic retreatment because the patient had already completed therapy, retreatment carries toxicity and resistance implications, and a mechanical postoperative explanation was present.

Negative microbiology does not fully exclude pediatric extrapulmonary TB, which can be paucibacillary and difficult to confirm [13]. In this case, however, the negative or non-confirmatory microbiology was not interpreted in isolation. It converged with bronchoscopy showing extrinsic rather than endoluminal disease, absence of surgically identifiable abnormal thoracic/hilar lymph nodes, pleural histopathology without granulomas or AFB, and imaging/operative evidence of a structural postoperative abnormality. The diagnostic question was therefore not whether a single test excluded TB, but whether the total evidence justified retreatment (Table 2).

**Table 2:** Multimodal evidence supporting or arguing against active tuberculosis in 2025 AFB: Acid-fast bacilli; CT: Computed tomography; MRI: Magnetic resonance imaging; PCR: Polymerase chain reaction; TB: Tuberculosis.

Domain	Finding	Supports active TB	Supports structural/postoperative mimic	Interpretation
Clinical symptoms	Fever, cough, weight loss, recurrent respiratory episodes	Yes, but non-specific	Non-specific	Raised suspicion but did not establish active TB
Prior Pott disease	Tissue-supported vertebral TB in 2021	Raises pretest suspicion	Explains postoperative anatomy and graft-related sequelae	Essential context, but not evidence of active 2025 disease
CT/MRI	Kyphotic gibbus, calcified mediastinal findings, intrathoracic bone-density material	Calcifications could prompt TB concern	Bone-density material adjacent to reconstruction favors graft-related complications	Imaging required operative and pathologic correlation
Bronchoscopy	Extrinsic tracheobronchial compression without ulcerated/granulomatous endoluminal lesion	Does not confirm active TB	Supports mechanical distortion/compression	Helped redirect reasoning away from active endobronchial TB
Microbiology	GeneXpert, AFB smears, cultures, and mycobacterial PCR negative or non-confirmatory	Does not fully exclude paucibacillary TB	Reduces probability when combined with other negative tissue findings	Insufficient alone; important in aggregate
Surgery	Pleuropulmonary adhesions, material compatible with prior graft, no abnormal thoracic/hilar nodes	No node target found	Directly supported structural/postoperative explanation	Exploration clarified the mismatch between the planned nodal biopsy and the actual pleural specimen
Pathology 2025	Fibrosing pleuritis without granulomas, caseation, active inflammation, AFB, or malignancy	Argues strongly against active pleural TB in the sampled tissue	Supports chronic/residual process	The key reason empirical retreatment was not restarted

The diagnostic strategy was selected because the consequences of error were substantial. Undertreatment of active TB could permit progression, whereas unnecessary retreatment could expose the child to hepatotoxicity, drug interactions, resistance-oriented consequences, and prolonged treatment burden. The operative approach was therefore justified only as an exceptional diagnostic step after less-invasive sampling was not documented as safe (Table 3).

**Table 3:** Diagnostic decision matrix for suspected tuberculous relapse after spinal tuberculosis reconstruction Evidence base: The management and procedural principles summarized here are supported by tuberculosis treatment guidance and pleural diagnostic literature [1-3,14-19]. ATT: Antituberculous therapy; VATS: Video-assisted thoracoscopic surgery.

Strategy	Advantages	Limitations	When to use	Application to this case
Percutaneous image-guided biopsy	Least invasive; can target pleura, node, or mass when safely accessible	Limited sample; risk if no safe trajectory; may miss heterogeneous disease	First-line tissue option when a safe target exists	Not documented as feasible because a safe window was absent or insufficient
VATS or medical thoracoscopy	High pleural diagnostic yield; less morbidity than open thoracotomy; allows pleural inspection and biopsy	May be limited by adhesions, distorted anatomy, airway risk, or availability	Preferred when pleural access is feasible and expertise is available	Not documented as selected; the record supports treating it as a theoretical alternative given no safe percutaneous window, altered anatomy, and operative adhesions
Open diagnostic thoracotomy	Direct visualization, larger tissue sample, evaluates adhesions and suspected graft-related mechanical problems	More pain, blood loss, recovery time, and anesthetic morbidity	Exceptional option when uncertainty is high, less-invasive routes are unsafe, and results will change management	Used to resolve whether retreatment for active TB was justified
Empirical ATT retreatment	Avoids delay when life-threatening or highly probable TB cannot be sampled	Toxicity, adherence burden, resistance concerns, and anchoring bias; may obscure alternative diagnoses	Consider if severe progression, high epidemiologic/clinical probability, and no diagnostic option exist	Avoided after negative/non-confirmatory microbiology, fibrosing pleuritis, and mechanical explanation
Observation/close follow-up	Avoids invasive procedures and unnecessary drugs	Unsafe if active TB, malignancy, or progressive compression is plausible	Appropriate when clinical risk is low and imaging is stable	Insufficient before surgery because uncertainty was clinically consequential

The radiologic abnormality in 2025 was most meaningful when interpreted against the 2021 operation. A bone-density intrathoracic/paravertebral structure near the prior corpectomy and reconstruction bed was not specific by imaging alone, but its location, density, and operative correlation made migrated graft material the most coherent explanation. The bronchoscopy finding of extrinsic compression further supported an anatomic effect rather than active endobronchial TB.

**Figure 2:**
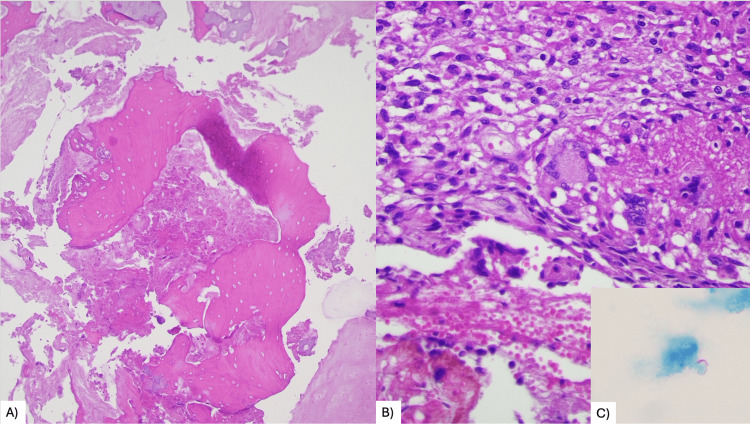
Histopathologic evidence supporting the original 2021 diagnosis of vertebral tuberculosis (A) Hematoxylin and eosin-stained vertebral/bony tissue section at 20x showing compact/trabecular bone with necrotic material. (B) Hematoxylin and eosin-stained section at 40x showing chronic granulomatous inflammation with Langhans-type multinucleated giant cells. (C) Ziehl-Neelsen stain at 100x was reported as positive for acid-fast bacilli. This figure supports the etiology of the original 2021 Pott disease; it does not represent the 2025 pleural biopsy. AFB: Acid-fast bacilli; H&E: Hematoxylin and eosin.

The 2021 vertebral histopathology is also central because it establishes that the original spinal disease represented true vertebral TB rather than a retrospective assumption. The available histology included necrotic bone, chronic granulomatous inflammation with Langhans-type giant cells, and Ziehl-Neelsen staining reported as positive for AFB (Figure 2).

By contrast, the 2025 pleural specimen showed fibrosing pleuritis without granulomas, caseous necrosis, active inflammation, AFB, or malignancy. Because the 2025 histology was non-granulomatous and essentially fibrosing, it is best described in the text rather than emphasized as a diagnostic image. Its main value was exclusionary: it did not support active pleural TB in the sampled tissue.

This report is not a formal systematic review; rather, it uses a focused narrative diagnostic review to organize the reasoning that made this case publishable. Structural mimics should be considered when symptoms recur after spinal TB reconstruction. Calcified mediastinal nodes or granulomas may reflect prior infection, while a bone-density structure adjacent to a reconstruction bed should prompt review of operative history before being interpreted as active granulomatous disease.

Less-invasive tissue acquisition remains the preferred diagnostic principle when technically safe. Pleural TB can require pleural tissue for diagnosis, and modern strategies favor image-guided pleural biopsy, medical thoracoscopy, or VATS when feasible [14-19]. Open diagnostic thoracotomy should be exceptional. In the available record, VATS was not documented as selected; therefore, the manuscript does not state that it was impossible. The open approach is framed as defensible because no safe percutaneous route was documented, postoperative anatomy was altered, pleuropulmonary adhesions were found intraoperatively, a graft-related mechanical complication required assessment, and the result determined whether prolonged antituberculous retreatment would be started (Table 4).

**Table 4:** Focused narrative review of pediatric spinal tuberculosis reconstruction and mechanical/diagnostic complications ADA: Adenosine deaminase; ATS: American Thoracic Society; CDC: Centers for Disease Control and Prevention; IDSA: Infectious Diseases Society of America; TB: Tuberculosis; VATS: Video-assisted thoracoscopic surgery; WHO: World Health Organization.

References	Population/focus	Key findings	Relevance to this case
WHO and ATS/CDC/IDSA guidance [1-3]	Pediatric and drug-susceptible TB management	Treatment decisions should consider severity, confirmation, susceptibility, and risk of delay.	Supports caution before empirical retreatment after prior completed therapy.
Garg and Somvanshi; Rajasekaran et al. [4,5]	Spinal TB concepts	Spinal TB may cause destructive vertebral disease, deformity, abscess, and neurologic risk.	Frames why the original 2021 disease required serious reconstruction and long follow-up.
Rajasekaran [6]; Rajasekaran et al. [7]	Pediatric deformity and surgical candidacy	Children may develop progressive kyphosis, and selected cases require surgery.	Supports the relevance of chronic gibbus and altered access anatomy.
Ozdemir et al.; Guo et al.; Wang et al. [8-10]	Allograft/fibular strut reconstruction in Pott disease	Structural grafts/allografts can restore anterior column support in selected cases.	Explains why fibular allograft reconstruction was a plausible initial strategy.
Lü et al. [11]	Thoracoscopy-assisted mini-open reconstruction	Less-invasive thoracic approaches have been used for thoracic spinal TB reconstruction.	Contextualizes the trend away from fully open approaches when feasible.
Wang et al. [12]	Graft migration/displacement after corpectomy	Graft displacement is a recognized mechanical complication after strut grafting.	Provides a spine surgery precedent for graft migration as a structural problem.
Seo et al. [13]	Pediatric extrapulmonary TB molecular diagnosis	Molecular tests may have limited sensitivity in extrapulmonary pediatric TB.	Prevents overclaiming that negative tests alone exclude TB.
Pleural TB diagnostic literature [14-19]	Pleural biopsy, thoracoscopy, ADA, and pleural TB management	Pleural tissue and less-invasive biopsy strategies are important in undiagnosed suspected TB pleuritis.	Supports the decision framework comparing percutaneous biopsy, VATS, thoracotomy, and empirical therapy.

Pediatric extrapulmonary TB may be paucibacillary, and negative molecular testing does not absolutely exclude disease [13]. Nevertheless, retreatment decisions should be anchored in the total clinical probability rather than reflexively in the past diagnosis. When microbiology, bronchoscopy, surgical findings, and histopathology all move away from active disease, the threshold for empirical retreatment should increase.

**Figure 3:**
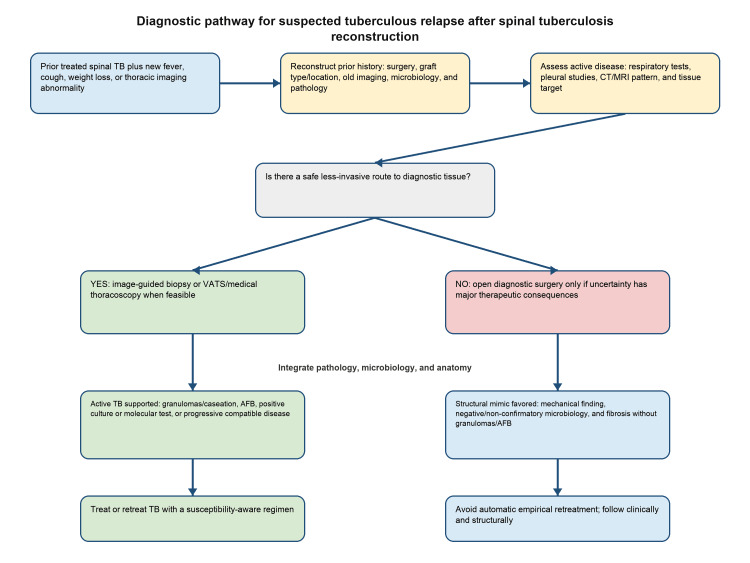
Diagnostic pathway for suspected tuberculous relapse after spinal tuberculosis reconstruction Proposed clinical decision pathway for children or adolescents with previous spinal TB reconstruction and later thoracic symptoms or imaging abnormalities. Less-invasive tissue diagnosis should be prioritized when safely feasible. Open diagnostic surgery is reserved for high-consequence uncertainty, unsafe less-invasive access, and a plausible mechanical complication. AFB: Acid-fast bacilli; TB: Tuberculosis; VATS: Video-assisted thoracoscopic surgery.

The proposed decision pathway is summarized in Figure 3. Its purpose is practical rather than prescriptive: it emphasizes that microbiology, pathology, imaging, operative history, and procedural feasibility should be integrated before labeling a postoperative structural abnormality as recurrent TB.

## Discussion

This case separates two diagnoses that could easily be conflated. The 2021 spinal disease was supported by immunologic, molecular, and histopathologic evidence consistent with vertebral TB. The 2025 thoracic presentation, however, remained a suspicion of pulmonary reinfection or pleural TB until tissue, microbiology, bronchoscopic documentation, imaging, and operative findings were integrated. The distinction matters because a history of TB does not make every subsequent calcified thoracic abnormality active disease.

The constitutional findings should be interpreted cautiously. Fever, weight loss, recurrent respiratory episodes, and severe malnutrition increased concern for active infection, but they were not specific for tuberculous relapse. The available records do not establish whether these symptoms were driven by non-tuberculous respiratory illness, chronic nutritional vulnerability, mechanical airway distortion, inflammatory response to postoperative sequelae, or a combination of factors. This uncertainty is part of the reason tissue correlation was necessary.

The intrathoracic bone-density material created a structural mimic with high diagnostic risk. In a child with prior Pott disease, fever, cough, weight loss, calcified mediastinal findings, and distorted postoperative anatomy, anchoring bias toward recurrent TB is understandable. The corrective step was not to dismiss TB but to require tissue correlation because the management consequence was substantial.

Bronchoscopic documentation contributed by showing extrinsic compression rather than an ulcerated, necrotic, or granulomatous endoluminal lesion. Surgery then clarified that the intended target of a thoracic/hilar node was not actually present as an abnormal node; the specimen submitted for pathology was right pleura. Pathology finally shifted the probability away from active disease by showing fibrosing pleuritis without granulomas, caseation, active inflammation, AFB, or malignancy.

PET-CT was not documented as performed in the available record. Although FDG PET/CT can help define the extent of extrapulmonary TB and may identify a metabolically active biopsy target, FDG uptake is not specific for active TB and may also occur with malignancy, postoperative inflammation, infection, or foreign-body reaction. Therefore, PET-CT would not have established active tuberculosis in this case; its main potential value would have been to identify an alternative, safely accessible biopsy site. Given the documented absence of a safe percutaneous window, altered postoperative anatomy, suspected graft-related mechanical complication, and the therapeutic consequence of prolonged retreatment, PET-CT would not necessarily have obviated the need for diagnostic thoracotomy [20].

The decision not to restart empirical antituberculous therapy was based on convergence rather than on a single negative test. Empirical therapy may be justified in severe or progressive suspected TB when the probability is high and tissue confirmation is impossible or unsafe. In this case, however, the availability of a mechanical explanation, negative or non-confirmatory microbiology, absence of abnormal thoracic/hilar nodes at surgery, and non-granulomatous pleural histology made automatic retreatment difficult to justify.

The migrated allograft also raises an orthopedic question distinct from the infectious diagnosis: how anterior column stability was maintained after a graft that originally served as structural support was no longer in its intended position. The reviewed records document chronic kyphotic deformity/gibbus and altered postoperative anatomy, but they do not include dynamic radiographs, a formal post-migration instability score, CT-based fusion assessment, or a documented explanation such as spontaneous auto-fusion. Therefore, residual spinal stability cannot be definitively characterized from the available record. The defensible clinical implication is that children with graft migration after spinal TB reconstruction require longitudinal spine follow-up to assess fusion mass, sagittal balance, kyphosis progression, neurologic risk, and the possible need for secondary stabilization [6,7].

Several biomechanical mechanisms could plausibly contribute to delayed allograft migration three to four years after surgery, including small-graft geometry, incomplete graft incorporation, remodeling during growth, chronic kyphotic loading, respiratory and thoracic motion, and pleuropulmonary adhesions within a prior thoracotomy field. These mechanisms remain hypotheses in this case because serial pre- and postoperative imaging suitable for mechanical reconstruction was not available. The point is not that one mechanism was proven, but that delayed graft migration is a credible structural explanation that must be separated from active TB recurrence [8-12].

Because this is a single-case report with a focused narrative diagnostic review, the findings should not be used to estimate the incidence or prevalence of intrathoracic allograft migration after pediatric Pott surgery. Source documents contained discrepancies regarding operative vertebral levels, so the reconstruction level was described conservatively as centered at T4. The interpretation of migration rests on clinical, imaging, documented bronchoscopic, operative, and histopathologic correlation rather than on a single definitive imaging sign. Direct pre- and postoperative comparison images demonstrating the graft before and after migration were not available in the manuscript media. The reviewed records also did not document final mycobacterial culture incubation duration, long-term post-intervention respiratory/orthopedic follow-up, dynamic spine imaging, formal instability scoring, or CT-based fusion assessment. These omissions limit conclusions regarding residual spinal stability and future kyphosis risk.

## Conclusions

Late intrathoracic migration of vertebral allograft material after pediatric Pott disease surgery can mimic pulmonary or pleural tuberculous relapse. In a previously treated child, calcified thoracic findings and constitutional symptoms should trigger careful evaluation, but not automatic retreatment. When microbiology is negative or non-confirmatory, bronchoscopic documentation suggests extrinsic compression, no abnormal thoracic or hilar lymph nodes are found surgically, and pleural histology shows fibrosis without granulomas, caseation, AFB, active inflammation, or malignancy, a structural postoperative mimic becomes the more defensible diagnosis. Diagnostic thoracotomy is exceptional, but it can be justified when safer biopsy routes are unavailable, and the result changes management. Because allograft migration may alter anterior column support, longitudinal spine follow-up is also necessary to document fusion, sagittal alignment, kyphosis progression, and the need for secondary stabilization.

## References

[1] (2026). WHO operational handbook on tuberculosis: module 5: management of tuberculosis in children and adolescents. https://www.who.int/publications/i/item/9789240046832.

[2] Nahid P, Dorman SE, Alipanah N (2016). Official American Thoracic Society/Centers for Disease Control and Prevention/Infectious Diseases Society of America Clinical Practice Guidelines: treatment of drug-susceptible tuberculosis. Clin Infect Dis.

[3] Saukkonen JJ, Duarte R, Munsiff SS (2025). Updates on the treatment of drug-susceptible and drug-resistant tuberculosis: an official ATS/CDC/ERS/IDSA Clinical Practice Guideline. Am J Respir Crit Care Med.

[4] Garg RK, Somvanshi DS (2011). Spinal tuberculosis: a review. J Spinal Cord Med.

[5] Rajasekaran S, Soundararajan DC, Shetty AP, Kanna RM (2018). Spinal tuberculosis: current concepts. Global Spine J.

[6] Rajasekaran S (2007). Buckling collapse of the spine in childhood spinal tuberculosis. Clin Orthop Relat Res.

[7] Rajasekaran S, Soundararajan DC, Reddy GJ, Shetty AP, Kanna RM (2023). A validated score for evaluating spinal instability to assess surgical candidacy in active spinal tuberculosis-an evidence based approach and multinational expert consensus study. Global Spine J.

[8] Ozdemir HM, Us AK, Oğün T (2003). The role of anterior spinal instrumentation and allograft fibula for the treatment of Pott disease. Spine (Phila Pa 1976).

[9] Guo Z, Yao M, Wang J, Sun K, Ji Z (2017). Treating children with spinal tuberculosis via debridement with allograft. West Indian Med J.

[10] Wang J, Zhang X, Zhang Y, Lv G, Wang X, Li J (2022). Posterior instrumentation combined with anterior debridement and reconstruction using allogenic strut bone for the treatment of children with multilevel lumbar spinal tuberculosis: minimum 5-year follow-up. BMC Musculoskelet Disord.

[11] Lü G, Wang B, Li J, Liu W, Cheng I (2012). Anterior debridement and reconstruction via thoracoscopy-assisted mini-open approach for the treatment of thoracic spinal tuberculosis: minimum 5-year follow-up. Eur Spine J.

[12] Wang JC, Hart RA, Emery SE, Bohlman HH (2003). Graft migration or displacement after multilevel cervical corpectomy and strut grafting. Spine (Phila Pa 1976).

[13] Seo YS, Kang JM, Kim DS, Ahn JG (2020). Xpert MTB/RIF assay for diagnosis of extrapulmonary tuberculosis in children: a systematic review and meta-analysis. BMC Infect Dis.

[14] Li C, Liu C, Sun B (2020). Performance of Xpert® MTB/RIF in diagnosing tuberculous pleuritis using thoracoscopic pleural biopsy. BMC Infect Dis.

[15] Gao S, Wang C, Yu X (2021). Xpert MTB/RIF ultra enhanced tuberculous pleurisy diagnosis for patients with unexplained exudative pleural effusion who underwent a pleural biopsy via thoracoscopy: a prospective cohort study. Int J Infect Dis.

[16] Roberts ME, Rahman NM, Maskell NA (2023). British Thoracic Society Guideline for pleural disease. Thorax.

[17] Zhou X, Jiang P, Huan X (2018). Ultrasound-guided versus thoracoscopic pleural biopsy for diagnosing tuberculous pleurisy following inconclusive thoracentesis: a randomized, controlled trial. Med Sci Monit.

[18] Aggarwal AN, Agarwal R, Sehgal IS, Dhooria S (2019). Adenosine deaminase for diagnosis of tuberculous pleural effusion: a systematic review and meta-analysis. PLoS One.

[19] Antonangelo L, Faria CS, Sales RK (2019). Tuberculous pleural effusion: diagnosis & management. Expert Rev Respir Med.

[20] Yu WY, Lu PX, Assadi M (2019). Updates on (18)F-FDG-PET/CT as a clinical tool for tuberculosis evaluation and therapeutic monitoring. Quant Imaging Med Surg.

